# Diet-microbiome-gut-brain nexus in acute and chronic brain injury

**DOI:** 10.3389/fnins.2022.1002266

**Published:** 2022-09-16

**Authors:** Maria Alexander Krakovski, Niraj Arora, Shalini Jain, Jennifer Glover, Keith Dombrowski, Beverly Hernandez, Hariom Yadav, Anand Karthik Sarma

**Affiliations:** ^1^Wake Forest University School of Medicine, Winston-Salem, NC, United States; ^2^Department of Neurology, University of Missouri, Columbia, MO, United States; ^3^Department of Neurosurgery and Brain Repair, University of South Florida, Tampa, FL, United States; ^4^Clinical Nutrition Services, Tampa General Hospital, Tampa, FL, United States; ^5^USF Center for Microbiome Research, Microbiomes Institute, University of South Florida, Tampa, FL, United States; ^6^Department of Neurology, Atrium Health Wake Forest Baptist, Winston-Salem, NC, United States

**Keywords:** microbiome, ketogenic diet, acute brain injury, chronic neurological disorders, stroke, neurodegeneration, probiotics

## Abstract

In recent years, appreciation for the gut microbiome and its relationship to human health has emerged as a facilitator of maintaining healthy physiology and a contributor to numerous human diseases. The contribution of the microbiome in modulating the gut-brain axis has gained significant attention in recent years, extensively studied in chronic brain injuries such as Epilepsy and Alzheimer’s Disease. Furthermore, there is growing evidence that gut microbiome also contributes to acute brain injuries like stroke(s) and traumatic brain injury. Microbiome-gut-brain communications are bidirectional and involve metabolite production and modulation of immune and neuronal functions. The microbiome plays two distinct roles: it beneficially modulates immune system and neuronal functions; however, abnormalities in the host’s microbiome also exacerbates neuronal damage or delays the recovery from acute injuries. After brain injury, several inflammatory changes, such as the necrosis and apoptosis of neuronal tissue, propagates downward inflammatory signals to disrupt the microbiome homeostasis; however, microbiome dysbiosis impacts the upward signaling to the brain and interferes with recovery in neuronal functions and brain health. Diet is a superlative modulator of microbiome and is known to impact the gut-brain axis, including its influence on acute and neuronal injuries. In this review, we discussed the differential microbiome changes in both acute and chronic brain injuries, as well as the therapeutic importance of modulation by diets and probiotics. We emphasize the mechanistic studies based on animal models and their translational or clinical relationship by reviewing human studies.

## Introduction

In recent years, growing attention has been placed on the microbiome playing a role in health and disease. The microbiome (also called “gut flora” and “gut”) can be defined as the presence of abundant living microorganisms (primarily bacterial organisms), their genes and their metabolically produced byproducts residing in the GI tract (Quigley, 2017). The microbiome influences physiology, metabolism, nutrition, and immune function; Specifically, the microbiota impacts host health by producing metabolites that affect physiology of host cells by manipulating cellular and molecular mechanisms (Li et al., 2018). Notable metabolites include short-chain fatty acids (SCFAs), the primarily colonic metabolites believed to be critical in neuronal signaling and function (Morrison and Preston, 2016; Dinan and Cryan, 2017), and trimethylamine *n*-oxide (TMAO), a metabolite produced by some bacterial strains linked to exacerbating cardiovascular disease and stroke risk (Zhu W. et al., 2021). Butyrate, the most common SCFA, is produced from butyrate-producing bacteria, which includes *Clostridium*, *Eubacterium*, and *Butyrivibrio* genera, and is associated with bacterial proliferation, colonocyte energetic supply, and brain therapeutic potential (Bourassa et al., 2016). A growing body of research is suggesting that the microbiome has a convincing and modulatory role in central nervous system (CNS) function and dysfunction (Yarandi et al., 2016).

The purpose of this article is to review the gut-brain relationship, evidence from both animal and human studies observing changes in microbiome signatures during acute and chronic neurological injury states, and the effect of the diets like the ketogenic diet (KD) in mitigating brain inflammation and supporting brain recovery. This review has the potential of serving as an evidence base to consider the use of microbiome modulators like KD, probiotics, and others in brain recovery and expand its use in acute brain therapy.

### The healthy gut

The colonization and character of the human microbiome is established at birth, with rapid development until 2 years old, after which much of the microbial composition is stabilized through adulthood with minor changes through aging (Fouhy et al., 2012). The initial and rapid colonization of the infant gut depends on several factors such as: mode of delivery, feeding type, antibiotic exposure, hospital environment, use of probiotics, and many more (Dominguez-Bello et al., 2010; Fouhy et al., 2012). The composition of the adult microbiome is largely dependent on regional/cultural diet and lifestyle practices (Biagi et al., 2011; Yatsunenko et al., 2012).

In the healthy state, a great amount of variation exists between each individuals’ microbiome profile, however overarching themes of strains exist. Bacterial phyla *Firmicutes* and *Bacteroidetes* are suggested to represent the largest proportion of microbiome (Yatsunenko et al., 2012), with microbial diversity maximized with a relative abundance of 80 and 15%, *Firmicutes* and *Bacteroidetes* respectively (Manor et al., 2020). Each individual’s enterotype is defined by the prominence of either *Prevotella*, *Bacteroides*, or *Ruminococcus* species (Arumugam et al., 2011). An individual’s enterotype is suggested to be affected by drug use and dietary choices (Arumugam et al., 2011; Wu et al., 2011).

In the healthy aging process, studies have determined a relatively low abundance of *Firmicutes*, and reduced bacterial diversity in the elderly population (Salazar et al., 2017). Metabolites of bacteria, notably SCFAs, are reduced in older age (Biagi et al., 2010; Salazar et al., 2013). Unfortunately, the elderly state is associated with a pro-inflammatory state (Schiffrin et al., 2010) with higher proportions of gram negative bacteria (Schiffrin et al., 2010) like *Enterobacteriaceae* and opportunistic bacteria like Proteobacteria, as well changes in the balance of microbes, all of which is linked to dysbiosis (Biagi et al., 2010; Shin et al., 2015).

The composition of the gut microbiome becomes unbalanced, altered, and in a state of “dysbiosis” due to a variety of reasons, including acute infection, acute and chronic disease, poor diet, and excessive antibiotic use (Vangay et al., 2015; Ballway and Song, 2021). Most clinical attempts to correct dysbiosis have been inadequate, but therapeutic utilization of fecal microbiota transplantation (FMT) has delivered successful results (Wang et al., 2019). FMT, often used to treat recurrent *Clostridium difficile* infections, involves transplanting stool from a healthy individual into another patient’s gastrointestinal (GI) tract to normalize the bacterial composition and correct a dysregulated gut (Surawicz et al., 2013; Gupta and Khanna, 2017; Wang et al., 2019). Long-term safety and efficacy of FMT in elderly and/or immunocompromised patients in treating severe, recurrent *Clostridium difficile* infections has been studied and proven successful (Kelly et al., 2014; Agrawal M. et al., 2016). Evidence for FMT provides an interesting perspective in disease treatment, especially correcting microbiome dysbiosis to relieve inflammation.

## Gut brain axis

**FIGURE 1:**
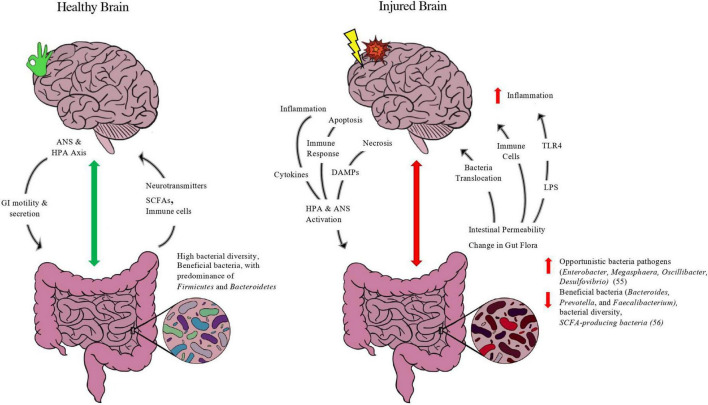
Purported model of gut-brain axis in healthy and injured brain. Depicted in figure is the bidirectional exchange between the brain and the gut in both a healthy state and injured state. In normal functioning, the brain’s neuroendocrine control of the gut through the autonomic nervous system (ANS) and hypothalamus-pituitary-adrenal (HPA) axis *via* the Vagus Nerve releases molecules such as acetylcholine and corticotropin-releasing hormone (CRH) to modulate enteric functions such as contractility, secretion of digestive enzymes, and immune function. A balanced microbiome with favorable features such as high bacterial diversity and abundance of beneficial taxa of bacteria such as firmicutes and short-chain-fatty-acid (SCFA) producing bacteria, such as *Roseburia* and *Faecalibacterium*, will exchange metabolites up the GBA. Gut bacteria produce neurotransmitters, such as serotonin, GABA, and dopamine, and SCFA-producing bacteria release SCFAs, metabolites directly linked to neuronal health and blood-brain-barrier integrity. The gut also is evidenced to modulate brain homeostasis and immune function by its release of macrophages and other white blood cells to aid in immune support. In an injured state, the interplay between the gut-brain is adjusted to meet the energetic changes and inflammation caused by brain injury. After harm is induced by trauma and/or compromised blood supply, the necrotic and injured brain tissue initiates apoptosis and inflammatory pathways that provoke the release of damage associated molecular patterns (DAMPs), cytokines, and other immune cells to trigger the ANS and HPA axis through the vagus nerve as well as sympathetic “stress” neuronal fibers. This injury response by the brain ensues changes to the gut, including microbial composition leading to dysbiosis and the predominance of opportunistic bacteria at the expense of more beneficial taxa, as well as increased gut epithelial barrier permeability. Such changes compromise the integrity of the gut (“leaky gut”), releasing bacteria and its metabolites up the GBA, including lipopolysaccharide (LPS), an endotoxin released from gram-negative bacteria that activates the transmembrane toll-like receptor 4 (TLR4) and initiates an innate immune response that exacerbates neuroinflammation. In addition, immune cells such as macrophages and neutrophils are released from the gut and migrate to the site of injury. While the migration of immune-fighting support can aid in the recovery after brain injury, it often aggravates the brain tissue and leads to delayed parenchymal recovery as well as secondary issues from prolonged inflammation.

At any given instant, there is bidirectional chatter between two brains: our central nervous system and our gastrointestinal system (our gut microbiome). The gut-brain axis (GBA) can be defined as the bidirectional communication between the gut and the CNS that is integrated with multiple signaling pathways, including the enteric nervous system, sympathetic and parasympathetic pathways of the autonomic nervous system, endocrine system, and immunological system (Wang and Wang, 2016). The bidirectional exchange of the GBA in healthy and injured states of the brain is depicted in Figure 1 below.

### Blood brain barrier

The brain, made of billions of neurons and glial cells, maintains connections to our body and gut in a selective and specific manner in a normal state of health. The brain is covered by a highly selective microvasculature called the blood brain barrier (BBB). The BBB is normally impermeable to many large molecules, polar substances, and bacteria through its tight junctions but allows passage of certain molecules through selective transport proteins on the BBB (Ballabh et al., 2004). The BBB can become disrupted by inflammation, such as during CNS hypoxic or injurious events that encourage the delivery of immunologic support to the brain and may contribute to the exacerbation of the CNS injury (Lochhead et al., 2017; Obrenovich, 2018).

During embryonic development of the BBB, the gut microbiome is hypothesized to be a contributor (Clarke et al., 2013) with evidence that the GBA is established from gestation (Braniste et al., 2014). Mice reared germ-free had more permeable BBBs than mice with a healthy microbiome and restoring the microbiome with SCFAs repaired BBB permeability to the levels of healthy pathogen-free mice (Braniste et al., 2014).

### Dysregulated gut-brain axis by central nervous system injury

There is crosstalk between the gut and brain along the GBA to maintain homeostasis and healthy functioning. The microbiome produces several key factors that contribute significantly to neuronal and glial cell function and metabolism, such as SCFA, Tryptophan metabolites and neurotransmitters, and vitamins (Fung et al., 2017; Pellegrini et al., 2020). The microbiome also interacts extensively with the peripheral immune system and engages in immune-bacterial interplay that affects the CNS (Pellegrini et al., 2020). Functions of the GI tract are modulated along the GBA, with evidence suggesting that vagal afferents communicate with brainstem nuclei to affect efferent fibers to modulate GI secretion and motility (Filpa et al., 2016).

Shortly after acute brain injury, ischemic tissue will propagate significant neuronal apoptosis, necrosis, and inflammation leading to the release of immune cells, damage associated molecular patterns (DAMPs), and cytokines along the GBA (Li et al., 2020). These down-stream inflammatory mediators lead to the activation of the autonomic nervous system and the hypothalamus-pituitary-adrenal axis (HPA) (Li et al., 2020) which initiate intestinal inflammation through the Vagus nerve (Filpa et al., 2016) and thought to increase intestinal permeability or “leaky gut.” Such that the GBA is a bidirectional relationship, the induced stress of the gut leads to changes in the gut microbiome and release of microbes, endotoxin lipopolysaccharide (LPS), immune cells, and inflammatory mediators like T cell populations (Th1, Th2, Th17, and Treg), and cytokines like tumor necrosis factor-alpha (TNF-α), IL-2, IL-12, IL-17 and interferon-gamma (IFN-γ) that further exacerbate inflammation (Shichita et al., 2009; Arya and Hu, 2018) up the GBA and into the bloodstream to lead to systemic inflammation (Wang and Wang, 2016; Arya and Hu, 2018). The release of LPS, endotoxin found on the outer membrane of gram negative bacteria, activates an innate immune response by binding Toll-like Receptor 4 (TLR4) (Kawai and Akira, 2010) and is linked to exacerbating brain injury, stroke development and neuroinflammation (Macrez et al., 2011; Klimiec et al., 2016; Kurita et al., 2020).

## Changes to brain-gut interactions during acute neurological injury

Acute neurological injuries such as ischemic stroke, traumatic brain injury (TBI), and intracerebral hemorrhages (ICH) are considered in this review. They each carry a high morbidity and mortality in the afflicted population. Understanding the brain-gut interactions in these acute injury states could lead to insights about possible new therapeutic interventions.

### Ischemic stroke and microbiome

Stroke is the fifth leading cause of morbidity and mortality in the U.S. (Herpich and Rincon, 2020). Ischemic stroke, which, due to compromised blood supply, leads to local brain damage, is followed by an immune and inflammatory response. The ischemic brain tissue releases inflammatory mediators and DAMPs such as nuclear protein high mobility group box protein 1 (HMGB1) and peroxiredoxin family proteins (Shichita et al., 2012) to initiate innate and adaptive immune responses through activation of TLR4 in the brain (Kurita et al., 2020). This is implicated in inducing LPS-mediated inflammation in the gut (Kawai and Akira, 2010) which in turn exacerbates neuroinflammation (Kurita et al., 2020). Cytokines, inflammatory mediators, and inflammatory cells from the gut are suggested to migrate to the injured brain (Wang and Wang, 2016). Several animal studies have identified migration of intestine-derived proinflammatory Th1 and Th17 cells to the injured brain (Benakis et al., 2016; Singh et al., 2016). In addition, as much as 50% of patients suffer from gastrointestinal complications with emerging research suggesting it to be related to a disrupted GBA after stroke (Camara-Lemarroy et al., 2014; Wen and Wong, 2017). Overall, there is strong support that ischemic stroke induces microbiome dysbiosis *via* top–down processes which affects bottom-up neuroinflammatory signaling from the gut (Tan et al., 2020).

#### Animal studies

In a rodent MCA occlusion model, post-stroke gut dysbiosis led to changes in bacterial composition, principally a decrease in species diversity and overgrowth of harmful *Bacteroidetes* and was related to increased gut barrier permeability and reduced gut motility following stroke (Singh et al., 2016). Another study observed a reduction in microbiota species and “stroke specific” changes in bacterial species 3 days after a cerebral artery occlusion (Yamashiro et al., 2017). These changes and preferential overgrowth of particular bacterial species has been hypothesized to be linked to reduced intestinal motility (Yamashiro et al., 2017), autonomic nervous system induced noradrenaline release (Houlden et al., 2016), and increased intestinal permeability (Meddings and Swain, 2000; Caso et al., 2009) inflicted by a post-stroke response. A proof-of-concept study in which healthy mice were transplanted with stroke-affected microbiomes observed increased Th1 and Th17 levels, as well as migration of T-cells to the brain (Yamashiro et al., 2017). Recently, enteric TMAO production has been directly linked to stroke severity by influencing cerebral infarct magnitude and adverse functional outcomes (Zhu W. et al., 2021).

#### Human studies

Changes in microbial composition has also been noted in human studies, including a study by Yin et al. (2015), that found stroke patients had higher proportions of opportunistic bacterial pathogens such as *Enterobacter, Megasphaera, Oscillibacter*, and *Desulfovibrio*, and a reduction in commensal or beneficial genera including *Bacteroides, Prevotella*, and *Faecalibacterium*. Similarly, another study found reduced levels of short chain fatty acid (SCFA)-producing bacteria (*Roseburia*, *Bacteroides*, *Lachnospiraceae, Faecalibacterium*, *Blautia*, and *Anaerostipes*) and enhanced levels of *Lactobacillaceae, Akkermansia*, *Enterobacteriaceae*, and *Porphyromonadaceae* in those with acute ischemic stroke (Tan et al., 2021). Reductions in SCFAs and SCFA-producing bacteria in stroke is associated with poorer outcomes 90 days after stroke (Tan et al., 2021). In another human study, microbial diversity between cerebral infarction patients and controls were comparable (Li H. et al., 2020); however, in cerebral infarction patients, levels of butyrate producing bacteria (BPB) were reduced while levels of lactic acid bacteria (LAB) increased. The study observed NIH Stroke Scale scores had a negative association with BPB and a positive correlation with LAB (Li H. et al., 2020). Overall, human and mice studies support microbial dysbiosis and exacerbation of inflammation after stroke.

### Traumatic brain injury and microbiome

Traumatic brain injury is the leading cause of death and disability in young adults in the western world (Winkler et al., 2016; Pearn et al., 2017) and contributes to nearly one-third of injury related deaths in the United States (Iaccarino et al., 2018). TBI is caused by a mechanical force that induces primary cerebral inflammation, including disrupting the BBB integrity that leads to edema, hemorrhage, hypoxia, cell death, axonal tearing, and gray–white matter disjunction secondary to impact forces (Blennow et al., 2012; Corps et al., 2015). BBB disruption, neural tissue damage, and disturbed autoregulation of cerebral blood flow has been associated with the post-traumatic epilepsy sequelae (Tomkins et al., 2011), increased ICP after TBI (Shahrokhi et al., 2010), and the release of immune “danger” signals (Manson et al., 2012), that can exacerbate neural injury (Corps et al., 2015) and induce systemic immune responses in peripheral organs (Tobin et al., 2014). TBI is also suggested to disturb the GBA by inducing GI mucosal barrier damage, leading to increased gut “leakiness” and mobilization of the defenses, activation of reactive gliosis, and entry of microbes into the CNS (Hanscom et al., 2021). Inflammatory migration to the brain is facilitated by acute BBB breakdown after TBI in humans (Hu and Tao, 2021). The systemic stress response after TBI activates the release of glucocorticoids by the Hypothalamic pituitary adrenal (HPA) axis and catecholamines by the ANS (Rosner et al., 1984; Koiv et al., 1997; Lemke, 2004; McDonald et al., 2020). Interestingly, gut dysbiosis has been reported to disrupt the HPA axis (Farzi et al., 2018), which prompts the question: does a dysregulated gut worsen post-TBI inflammation? The microbiome profile in individuals with TBI and other neurodegenerative diseases compared to normal controls and their respective animal models are vastly different (Gorjifard and Goldszmid, 2016; Ma et al., 2017, 2019; Shen et al., 2017; Simon et al., 2017; Vogt et al., 2017; Grosicki et al., 2018; Kigerl et al., 2018; Treangen et al., 2018; Zhan et al., 2018; Zhu et al., 2018; Zhuang et al., 2018; Nagpal et al., 2019; Rice et al., 2019; Royes and Gomez-Pinilla, 2019; Adriansjach et al., 2020; Urban et al., 2020). Several animal and human studies have studied the relationship between TBI and the microbiome.

#### Animal studies

In a systematic review by Pathare et al. (2020) seven studies discussing the impact of TBI on the gut microbiome were evaluated, six of which were animal studies. All studies determined a change in microbiome composition, especially an enrichment in *Proteobacteria* and *Firmicutes* bacterial populations across all studies. Interestingly, a study that analyzed rodents’ microbiome 2 h after moderate TBI identified that the volume of injured parenchyma correlated positivity with *Proteobacteria and* negatively with *Firmicutes* levels (Nicholson et al., 2019). In a more recent study using a mouse model, it was noted that microbiome diversity had time-dependent changes from 1 h to 7 days post-TBI. The study noticed TBI-induced gut inflammation was related to depleted levels of bile acids and suggested that changes to bile acid metabolism may be played by *Staphylococcus* and *Lachnospiraceae* (You et al., 2022).

#### Human studies

A recent human study with 101 moderate-severe TBI patients confirmed the enrichment of *Proteobacteria* in TBI as seen in previous rodent models, with the largest group being *Enterobacteriaceae*, as early as 48 h after impact (Mahajan et al., 2021). A study observed the changing microbiome profile of critically injured patients in which 8 of the 12 participants sustained a TBI (Howard et al., 2017). Researchers determined that there was no observed difference in microbial diversity between injured and non-injured patients upon ED admission; however, changes to the microbiome composition were dramatic within 72 h with a reduction of bacterial orders *Bacteroidales, Fusobacteriales*, and *Verrucomicrobiales* and enrichment of *Clostridiales* and *Enterococcus* in trauma patients (Howard et al., 2017). The increased ratio of pathogenic to commensal bacteria in the gut after trauma is thought to encourage the progression of post-TBI disease severity (Pathare et al., 2020). In a recent case-control study, researchers noted a significant increase in the composition *of Enterococcus, Parabacteroides, Akkermansia*, and *Lachnoclostridium* and a reduction in the abundance of *Bifidobacterium* and *Faecalibacterium* in TBI patients (Hou Y. et al., 2021). In summary, recent literature is convincing that TBI severity and a dysregulated gut composition are intertwined.

### Intracerebral hemorrhages and microbiome

Compared to the above neurological injuries, hemorrhagic strokes or “brain bleeds” have less supporting data regarding their effect on the microbiome. Cerebral hemorrhages include subarachnoid hemorrhages (SAH) from trauma and aneurysmal rupture, intraparenchymal hemorrhage (IPH), subdural hemorrhage (SDH), and epidural hemorrhage (EDH). ICH accounts for 2.8 million deaths per year (Benjamin et al., 2019) and the poor outcome after ICH is due to the secondary injury and neuroinflammation propagated by the primary hematoma (Zhao et al., 2014; Duan et al., 2016; Zhu et al., 2019; Yu et al., 2021b). The detrimental effects of ICH are largely secondary to the induced neuroinflammation which is suspected to be related to proinflammatory T cell production of cytokines and increase in BBB permeability (Arumugam et al., 2005).

#### Animal studies

To observe the effect of the GBA and microbiome on T cell regulation after ICH, a recent rodent study by Yu et al. (2021b) observed that ICH altered the gut integrity and impaired gut motility, and T cells from the intestines migrated to perihematomal areas and exacerbated ICH neuroinflammation. Interestingly, researchers observed that FMT-treated mice had lower levels of IFN-γ, IL-17, and mRNA expression levels of TNFα 2 weeks following the injury (Yu et al., 2021b), suggesting that the gut microbiome has a strong role in neuroinflammation after ICH. In addition, FMT after ICH significantly alleviated the ICH-induced neurobehavioral deficits and restored gut barrier integrity (Yu et al., 2021b).

Another study using a rat model noted an increase of gut permeability from onset to 7 days following ICH-induction as well as a reduction in IgA and gut barrier tight junction markers (Zhang et al., 2021). The study found that the microflora composition of the ileum and lungs were similar, suggesting the translocation of enteric microflora to the lungs which prompted a pulmonary infection after hemorrhage (Zhang et al., 2021). Moreover, researchers noted that aggravation of the gut permeability promoted the migration of intestinal bacteria and increased the risk of post-injury pneumonia (Zhang et al., 2021). Hemorrhagic transformations (HT) following stroke were analyzed in mice and linked to an elevation in *Proteobacteria* and *Actinobacteria* bacteria and reductions in SCFAs. Exacerbation of HT was not seen in mice treated with antibiotics suggesting that susceptibility to HT may be related to changes to the microbiome (Huang et al., 2022).

Subarachnoid hemorrhage (SAH), is most often caused by a ruptured aneurysm (Petridis et al., 2017). In a SAH rodent model, a study found an interesting link between microbiome depletion and aneurysm formation: antibiotic use significantly reduced the risk of aneurysm formation, as well as reduced infiltration of inflammatory markers (Shikata et al., 2019). Overall, the results from recent animal studies support that the outcome as well as potentially the predisposition to cerebrovascular hemorrhages may be related to the microbiome.

#### Human studies

More recent literature has suggested a link between ICH and the gut. High plasma concentrations of TMAO have been demonstrated to correlate with poor 3-month function outcomes in human ICH patients (Zhai et al., 2021) and low levels of butyrate-producing bacteria was found to be associated with hemorrhagic strokes (Haak et al., 2021). FMT therapy also had efficacy in a human ICH case. A case reported a male patient developing sepsis and multiple organ dysfunction syndrome (MODS) after initial admission for a cerebellar hemorrhage, and successfully underwent a FMT to alleviate MODS (Wei et al., 2016). The FMT successfully corrected the dysbiosis by profoundly enhancing the abundance of *Firmicutes* and depleting *Proteobacteria* (Wei et al., 2016). Beyond this data, limited studies have evaluated the acute occurrence of brain bleeds with changes to the microbiome in humans.

## Changes to brain–gut interactions during chronic neurological injury

The gut microbiome’s role in the development of chronic neurological disorders such as Alzheimer’s disease, Parkinson’s disease, epilepsy, vascular dementia, and primary brain tumors is discussed in this review. Interestingly, while the mechanisms of GBA disruption in acute and chronic brain injury have distinctions, there are considerable parallels to appreciate.

### Alzheimer’s disease and microbiome

Alzheimer’s disease (AD) is a neurodegenerative disease with a state of chronic neuroinflammation and synaptic dysfunction as a primary manifestation (Lepeta et al., 2016; Schain and Kreisl, 2017; Bairamian et al., 2022) and its’ pathogenesis is attributed to several unelucidated comorbid neuropathologies (Soria Lopez et al., 2019).

Recent literature in both rodent and human studies have observed evidence of gut microbiome dysbiosis in advanced AD, suggesting a significant role of the microbiome in the pathogenesis of the AD (Bairamian et al., 2022).

#### Animal studies

In a rodent pre-clinical model, a role of the microbiome in Abeta amyloid development was suggested (Harach et al., 2017). The sequenced microbiome of Aβ precursor protein (APP) transgenic mice was vastly different than wild-type mice, with significant reductions in *Firmicutes, Verrucomicrobia, Proteobacteria*, and *Actinobacteria* and simultaneous increases in *Bacteroidetes* and *Tenericutes* phyla (Harach et al., 2017). Moreover, germ-free APP mice have greater increases in Aβ levels after FMT from conventionally reared APP mice compared to FMT from wild-type mice (Harach et al., 2017). Another rodent study links the production of Aβ proteins with the host microbiome (Minter et al., 2016); broad-spectrum antibiotic use shifted the microbial composition of rodents, including enhancing genus *Akkermansia* and family *Lachnospiraceae*, reduced cerebral Aβ deposits, and decreased neuro-inflammatory reactive gliosis to Aβ plaques (Minter et al., 2016). More recently, a study using a rodent AD model with gut dysbiosis linked increased expression of gut NLRP3 to the activation of peripheral inflammation and exacerbation of AD neuroinflammation (Shukla et al., 2021), providing a potential mechanism between gut dysbiosis induced AD progression.

#### Human studies

In human patients, increases in the abundance of pro-inflammatory taxa like *Escherichia/Shigella* and decreases in anti-inflammatory taxon *E. rectale* was observed in the stool of mildly impaired patients and associated with increases in inflammatory cytokines IL-1β, NLRP3, and CXCL2 and brain amyloidosis (Cattaneo et al., 2017). In another study, the bacterial taxonomic makeup of AD revealed lower bacterial diversity and a remarkable shift in bacteria composition, with decreased *Firmicutes*, increased *Bacteroidetes*, and decreased *Bifidobacterium* in those with AD compared to a distinctly different profile in age-matched controls (Vogt et al., 2017). It is thought that the abundance of the pathogenic gram-negative bacteria *Bacteroidetes* may increase the release of LPS into systemic inflammation, initiating an immune response that exacerbates neuroinflammation in AD (Bairamian et al., 2022).

The study also drew a positive correlation between the severity of microbiome change and the abundance of AD biomarkers in the cerebral spinal fluid (CSF) (Vogt et al., 2017). Another study observed a relation between AD and microbiome alterations, with *Faecalibacterium prausnitzii* correlating with less cognitive impairment in patients and that the administration of isolated strains of the bacteria improved cognitive functioning in a rodent model (Ueda et al., 2021). Interestingly, different microbiome signatures were observed within those with mild cognitive impairment (MCI); a study correlated levels of *Proteobacteria* with Aβ-42: Aβ-40 while fecal levels of propionate and butyrate negatively correlated with Aβ-42 (Nagpal et al., 2019). All taken together, the current state of literature is strong in its link between the progression of AD, gut dysbiosis, and the subsequent neuroinflammation, as well as the emerging potential of modulating the microbiome in approaching AD management (Bairamian et al., 2022).

### Parkinson’s disease and microbiome

Parkinson’s disease (PD) is a neurodegenerative movement disorder due to the dramatic reduction of dopaminergic neurons in the substantia nigra. Interestingly, the pathological marker of PD, alpha-synuclein aggregates, is first detected in the enteric nervous system prior to the discovery in the brain (Felice et al., 2016), highlighting a role in the GBA in the progression of PD. To this end, researchers have found a suggestive link between PD progression and gut dysbiosis.

#### Animal studies

Recently, a study found that Osteocalcin (OCN), an osteoblast-derived protein, was successful in reducing dopaminergic cell loss and motor deficits in a PD mouse model through modulation of the gut; OCN depleted *Firmicutes* and increased abundance of *Bacteroidetes*, and OCN’s neuroprotective effect is thought to be facilitated by propionate, a SCFA (Hou et al., 2021).

#### Human studies

Sequencing of PD patients’ fecal samples indicated 77.6% lower levels of *Prevotellaceae than the levels in* age-matched controls (Scheperjans et al., 2015), significant in that *Prevotellaceae* is associated with maintaining gut barrier integrity through its role in mucin synthesis (Zhu X. et al., 2021). A high prevalence of *Enterobacteriaceae* was noted in PD as well as a strong correlation with worse impairments in postural instability and gate performance in PD patients (Scheperjans et al., 2015). Another PD patient study indicated a higher abundance of pro-inflammatory *Proteobacteria* and lower abundance of commensal *Faecalibacterium* and butyrate-producing bacteria (Keshavarzian et al., 2015). In short, the microbiome’s influence on the GBA and the pathogenesis of PD is an unfolding topic.

### Epilepsy and microbiome

Epilepsy, a chronic neurological condition predisposing patients to frequent seizure production in the brain, estimates 2.4 diagnoses each year (Lum et al., 2020). Seizures consist of disbalanced neuronal excitation by glutamate and are prompted by various neurochemical, ionic, or traumatic triggers (Lum et al., 2020). Currently, anti-epileptic drugs (ADEs) have provided tremendous support for a number of patients with epilepsy, while many others are drug-resistant and have “refractory epilepsy.”

#### Animal studies

Nearly 15 years ago, a rodent study established a correlation between peripherally stimulated intestinal inflammation and increased neuroinflammation and seizure susceptibility (Riazi et al., 2008). In a more recent rodent model of epilepsy, while intestinal inflammation was associated with ineffective ADE responses and increases in seizure activity, administration of SCFA butyrate had anticonvulsant effects (De Caro et al., 2019). In a study of the microbiome with seizure susceptibility, both rats subjected to chronic stress as well as healthy rats that underwent FMT of stressed mice had accelerated development and prolongation of seizures (Medel-Matus et al., 2018). Animal studies have established a suggestive relationship between dysbiosis and seizure likelihood, and further research has extended to human subjects.

#### Human studies

Three human subject trials have observed altered gut composition with increased levels of *Firmicutes* with relation to *Bacteroides* in patients with refractory epilepsy (Xie et al., 2017; Peng et al., 2018; Lindefeldt et al., 2019). A recent review comparing the three above studies reports that there is not a clear understanding on the alpha diversity change in refractory epilepsy (Lum et al., 2020). Antibiotic use has been associated with changes in seizure risk; a meta-analysis of carbapenem use illustrated an enhanced seizure risk (Cannon et al., 2014) while a combination treatment of macrolides with a penicillin derivative was observed to render a small cohort of patients seizure-free for a period of time (Braakman and van Ingen, 2018). In patients with drug resistant epilepsy, the KD has been known to be therapeutic, and researchers have recently identified a link to the microbiome, which is discussed in the “Ketogenic Diet” section.

### Vascular dementia and microbiome

Vascular dementia (VaD), caused by diminished blood supply to the brain due to a compromised cerebrovascular system, is the second most common cause of dementia and leads to a progressive cognitive, functional, and memory impairments (Sahathevan et al., 2012).

#### Animal studies

Due to the evidence of bidirectional signaling along the GBA, several studies have attempted to better understand the role of the microbiome in the pathogenesis of VaD, but they are limited to rodent studies. A study by Liu J. et al. (2015) was the first to find a modulatory effect on VaD by the microbiome (Sahathevan et al., 2012). Production of butyrate by *Clostridium butyricum* was shown to have a neuroprotective effect against VaD in a mouse model through improvement of cognitive function, reduced apoptotic rate, and decreased histological changes in the hippocampal CA1 region (Liu J. et al., 2015). In addition, mice models treated with *Clostridium butyricum* increased levels of Brain-derived neurotrophic factor (BDNF), a potent neural mediator of inflammation and apoptosis (Fanaei et al., 2014). The pathogenesis of VaD is growing in its understanding, and a role by the microbiome is considered but is in need of further study (Mirzaei et al., 2021).

### Brain tumors and microbiome

Brain cancers, composed of tumors in the central nervous system, are defined by a chronic state of proliferative growth and angiogenesis. A recent review by Mehrian-Shai et al. (2019) has proposed mechanisms in which the progression of brain cancers can be encouraged by the microbiome and its interplay with host immune function and inflammation, but microbiome studies are mostly limited to a subset of primary brain cancers, gliomas. Glioma, a type of CNS tumor, is the leading cause of brain-related cancer deaths (Weller et al., 2015). While the etiology of gliomas are not clear, recent advances have suggested a relationship between the gut microbiome and its metabolites in facilitating the protective effects of anti-cancer agents (Dono et al., 2022).

Understanding of the microbiome in brain tumors is sparse, and research into microbial changes are limited primarily to animal studies which have recently acknowledged a link between glioma formation and microbiome dysbiosis. In a recent study, Fan et al. (2022) determined that glioma growth changed the gut microbiome, namely by increasing the abundance of *Firmicutes* and decreasing the abundance of *Bacteroidia*, in a rodent model (Fan et al., 2022). In addition, researchers observed that gut dysbiosis encouraged glioma growth and downregulation of cerebral Foxp3 expression (Fan et al., 2022), a regulator of regulatory T cell function, which emphasizes a bidirectional influence on the gut and brain through communications along the GBA.

A study of human glioma patients and mouse models found that certain neurotransmitters and SCFAs (butyrate, acetate, and propionate) were reduced with glioma (Dono et al., 2020). Researchers also observed that animal models on temozolomide (TMZ), a chemotherapeutic, did not develop the microbiota alterations associated with glioma (Dono et al., 2020). Another study evaluating the effect of glioma and TMZ on the gut observed that TMZ alleviated the changes seen in glioma-bearing mice; without the effect ofTMZ on the microbiome, fecal samples from glioma-bearing mice and human glioma patients had similar patterns of altered *Firmicutes*: *Bacteroidetes* and increased pathogenic *Verrucomicrobia* phylum and *Akkermansia* genus compared to control mice and patients (Patrizz et al., 2020). These two studies provide an interesting perspective on how a cytotoxic chemotherapeutic like TMZ has a strong modulatory effect on the microbiome and may provide an alternative mechanism in which TMZ mitigates glioma formation.

Another rodent study administered antibiotics and observed increased glioma growth, altered gut composition, reduced cytotoxic natural killer cells, and altered microglia expression (D’Alessandro et al., 2020). Preliminary pre-clinical studies in glioma-bearing mice have observed that gut microbiome taxa, namely *Akkermansia muciniphila*, might affect the response of PD-L1, an immune checkpoint (Dono et al., 2022). *Akkermansia muciniphila* has had evidence in mitigating the PD-L1 response in other non-brain cancers (Sivan et al., 2015; Routy et al., 2018). While still young is the study of glioma and the microbiome, results from several studies provide a plausible relationship between glioma and dysbiosis, potential mechanisms in which drugs like TMZ can affect glioma formation through affecting the microbiome, and a potential effect of microbial organisms such as *Akkermansia muciniphila in* affecting the progression of cancer.

### Amyotrophic lateral sclerosis and microbiome

Amyotrophic lateral sclerosis (ALS) is a progressive neurodegenerative disease that impacts upper and lower motor neurons, compromising functions such as breathing and ultimately leading to death typically within 2 years of disease onset. While some cases of ALS have been linked to genetic mutations such as *SOD1*, many underlying causes of ALS and how they affect prognosis is not yet elucidated (Chio et al., 2009; Boddy et al., 2021). Attention on the effect of the microbiome on ALS disease progression and severity has been considered in animal and human models (Zhang et al., 2017).

#### Animal studies

In mouse models, convincing correlations with microbiome health and ALS severity have been made. In a study by Zhang et al. (2017) SOD1^G93A^ mice supplemented with butyrate observed improved survival, enhanced gut barrier integrity and replenishment of butyrate-producing bacteria in the gut. Another study with SOD1^G93A^ mice correlated 11 bacteria with disease severity, such as *Ruminococcus torques* and *Parabacteroides distasonis*, and indicated a protective effect with treatment of *Akkermansia muciniphila* (Blacher et al., 2019). A recent study using *C9orf72*^Harvard^ mice observed prevalence of *Helicobacter* spp. in mouse models, and the administration of antibiotics decreased the abundance of *Helicobacter* spp. as well as the emergence of cytokine storms, neutrophilia, or other inflammatory phenotypes (Burberry et al., 2020).

#### Human studies

Several human studies noted low *Firmicutes*/*Bacteroidetes* ratio in ALS patients (Fang et al., 2016; Rowin et al., 2017; Zeng et al., 2020). Interestingly, a recent study linked an increase F:B ratio and higher bacterial diversity with accelerated disease progression in patients with Motor Neuron Disease (MND) (Ngo et al., 2020). One study also noted significantly greater abundance of harmful organisms within genus *Dorea* and reductions of beneficial bacteria like *Oscillibacter*, *Anaerostipes, Lachnospiraceae* (Fang et al., 2016). In addition, a study noted low microbial diversity in all five ALS patients and reduced levels of beneficial *Ruminococcus* in majority of ALS patients with reduced F/B levels (Rowin et al., 2017). A longitudinal study observing ALS patients’ microbiomes in between two time points noted significant fluctuations in microbial composition, such as increase in pro-inflammatory *Cyanobacteria* (Di Gioia et al., 2020). Human studies suggest a strong connection between dysbiosis and ALS and has led to in the study of probiotic supplementation in disease therapy (Di Gioia et al., 2020). While the study of 6-month probiotic supplementation suggested no substantial changes to the microbial composition of ALS patients (Di Gioia et al., 2020), interest in microbiome modulation and ALS disease progression is strong.

### Multiple sclerosis and microbiome

Multiple Sclerosis is a neuroinflammatory disease, with acute and chronic exacerbations, that attacks the CNS. Insults lead to inflammatory lesions in the CNS and symptoms of fatigue, vision changes, and motor functions. The etiology of MS has been related to several factors, such as genetics, obesity, viral infections, and more recently, the microbiome (Preiningerova et al., 2022).

#### Animal studies

The microbiome’s involvement in immune dysregulation and disease development has been studied in the context of relapse-remitting MS for over a decade. In a relapsing-remitting mouse model of spontaneously developing experimental autoimmune encephalomyelitis (EAE), researchers suggest that the initiation of an endogenous immune response is dependent on commensal gut flora (Berer et al., 2011), and the recruitment of autoantibodies rely on the dual role of gut flora and myelin oligodendrocyte glycoprotein (Berer et al., 2011). Additional rat models of EAE suggest a link between microbial dysbiosis with MS progression. Studying colonizing EAE mice with beneficial bacteria has suggested improvement in disease severity; *Bacteroides fragilis* has been linked to protecting the susceptibility to EAE (Ochoa-Reparaz et al., 2010) and human-derived bacteria *Prevotella* suppressed the activation of Th1 and Th17 cells and mitigated inflammation in EAE (Mangalam et al., 2017). In contrast, establishment of segmented filamentous bacteria (SFB) in mice guts promotes the inflammatory IL-17 response and worsens EAE (Lee et al., 2011).

#### Human studies

When comparing the microbiome between identical twins discordant for MS, the MS patients had overall reduced diversity and significant increases in harmful taxa like *Akkermansia* (Berer et al., 2017). Interestingly, the MS patients’ stools were transplanted into mice and led to a higher incidence of spontaneous brain autoimmunity (Berer et al., 2017). In pediatric patients with relapsing-remitting MS (RRMS), shorter relapse intervals were significantly associated with reduction of *Fusobacteria* and expansion of *Firmicutes* (Tremlett et al., 2016). Studies have correlated dysbiosis of the microbiome in RRMS with observed enrichment of harmful genera *Psuedomonas, Mycoplana, Haemophilus, Blautia*, and *Dorea* (Chen et al., 2016) and reduction of SCFA-producing bacteria in MS patients (Takewaki et al., 2020). Taken together, several studies note disadvantageous changes to the gut microbiome in patients with MS.

## Dietary interventions on brain injury

### Probiotic supplements and diet

Probiotics are defined as live strains of selected bacteria (Hill et al., 2014), present in foods such as yogurt, kefir, kombucha, miso, and other fermented foods, that supplement the composition of consumers’ microbiomes. The study of supplementary probiotics’ relation to brain injury is limited, with human studies mainly in the study of drug-resistant epilepsy. In a recent 2018 pilot study, the use of an eight-strain probiotic, including the three genres of *Lactobacillus*, *Bifidobacterium*, and *Streptococcus*, in 45 drug resistant epilepsy patients noted significant improvement in quality of life and 50% reduction in the number of seizures in 28.9% of patients, leading authors to suggest the safe use of probiotics in mitigating seizures (Gomez-Eguilaz et al., 2018). In a study of neonates with rotavirus in a Neonatal Intensive Care Unit (NICU) setting, administration of probiotics, namely *Saccharomyces boulardii* or *Lactobacillus casei*, within 24 h of birth reduced the odds ratio of developing seizures while in the NICU (Yeom et al., 2019). Probiotic use in a small cohort of MS patients suggested modest success in decreasing the release of inflammatory cytokines such as intermediate monocytes (CD14^high^CD16^low^) and CD80 monocytes, and increased abundance of taxa, notably *Lactobacillus*, S*treptococcus*, and *Bifidobacterium* species (Tankou et al., 2018a). Another study suggested a synergistic effect of probiotics and current MS therapies, with probiotic administration linked to increases in depleted taxa like *Lactobacillus* and reduced the abundance of MS-associated taxa *Akkermansia* and *Blautia* (Tankou et al., 2018b).

The use of probiotic supplementation has been studied in stress-induced animal models and has some evidence in mitigating symptoms of anxiety and depression through various unelucidated potential mechanisms, including microbial composition alterations, reduction in cytokines, increase in brain-derived neurotrophic factor, and modulating inflammatory immune pathways (Liu X. et al., 2015). Probiotic supplementation in stroke was recently reviewed (Wu and Chiou, 2021), with studies limited to animal models that suggested beneficial protection and improved ischemia with probiotic use, such as a study that observed mitigation of infarct volume and neuronal cell death with *Lactobacillus amylovorus* DSM 16698T (ILA) supplementation (Wanchao et al., 2018) and another study that noted significant reduction in tumor necrosis factor-alpha level with *Bifidobacterium breve, Lactobacillus casei, Lactobacillus bulgaricus*, and *Lactobacillus acidophilus* supplementation (Akhoundzadeh et al., 2018). Supplementation of *Lactobacillus* spp. to a rodent model of MS mitigated neuroinflammation and correlated to IL-10’s production of regulatory T cells (Lavasani et al., 2010).

A recent review on the Mediterranean diet addressed several human studies that considered fish intake as neuroprotective against dementia and cognitive decline, as well as several human studies that correlated lower stroke risk with fish intake (Roman et al., 2019). Researchers related the mechanisms behind the observed health benefits to an increase of plasma omega-3 fatty acids after fish consumption (Roman et al., 2019). Regarding diet and acute brain injury, whole grain consumption through cold breakfast cereal and bran has been linked to significantly lower ischemic stroke incidence in two cohort studies (Juan et al., 2017). The effect of different diets on the outcomes in acute brain injury are described in Table 1. The therapeutic potential of a specific dietary intervention is particularly promising in the realm of brain injury: the ketogenic diet.

**TABLE 1:** Summarized findings of diet on brain injury and neuroinflammation.

Diet	Source	Neural injury	Model	Methods	Findings
Fructose	Agrawal R. et al., 2016	TBI	Male Sprague -Dawley rats	Eight weeks of fructose drink	Fructose disrupted hippocampal mitochondrial, cell homeostasis and plasticity, worsened spatial memory, and promoted membrane lipid peroxidation.
High-fat sucrose (HFS)	Hoane et al., 2011	Cortical contusion	Sprague–Dawley rats	Eight weeks HFS diet prior to bilateral frontal cortical contusion injuries (CCI)	HFS-fed mice had worsened performance on somatosensory and memory tasks and greater loss of parenchyma post-CCI compared to standard diet.
	Wu et al., 2003	TBI	Male Sprague -Dawley rats	Four weeks of HFS diet prior to mild fluid percussion injury or sham surgery	HFS further aggravated the FCI-induced impairment of spatial learning and cognitive function; suggested to be due to HFS’s reduction of brain-derived neurotrophic factor.
High-fat	Sherman et al., 2016	Mild TBI	C57 BL/6 Mice	60% kcal HFD for 4 months, deemed obese at 25–30% weight gain, then TBI *via* controlled skull impact (CSI) device.	30 days after injury, heightened microglial activation was present in injured HFD mice. Also noted male obese mice had worse outcomes than obese female mice.
	Puig et al., 2012	TBI	C57 BL/6 Mice	22-week HFD (21.2% by weight)	HFD-mice demonstrated enhanced NF-α, microglia, and macrophage activation in the brain and adipose tissue as well as circulating amyloid precursor proteins.
High tryptophan (HTD)	Yue et al., 2021	SUDEP	DBA/1 mice	Mice experiencing seizure-induced respiratory arrest (S-IRA) assigned to HTD or normal	HTD significantly reduced the rates of S-IRA, levels of cranial 5-HT and 5-HIAA, and increased microbiome diversity. There was increased composition of *Proteobacteria* and *Actinobacteria* after HTD.
High Fiber	Matt et al., 2018	Neuro-Inflammation in aged mice	Aged mice	1% cellulose (low fiber) or 5% inulin (high fiber) diet for 4 weeks	Mice after high fiber diet consumption had reduced microglial pro-inflammatory gene expression as well as inflammatory infiltrates in the colon. High fiber diet altered the gut microbiome composition and increased SCFA production, especially butyrate and acetate.
Mediterranean Diet (MeD)	Estruch et al., 2018	Ischemic stroke	High cardiovascular risk Humans	Participants split into: (1) MeD supplemented with extra virgin olive oil, (2) MeD supplemented with nuts, (3) control group with low-fat diet	Lower rates of major cardiovascular events occurred in participants assigned to an energy-unrestricted Mediterranean diet, supplemented with extra-virgin olive oil or nuts, compared to those assigned to a reduced-fat diet.
High fruit and vegetable diet	Aune et al., 2017	Stroke	Healthy humans followed for stroke incidence	Various methods – (meta analysis). Most followed patients for stroke risk	Relative risk reduction of stroke with vegetable and fruit intake – 20% RRR with 200–350 g/day, 28% RRR with 500 g/day, and 33% RRR with 800 g/day.

### Ketogenic diet

The KD is defined as a high-fat, adequate-protein, low-carb diet (Pondel et al., 2020). The KD originates from science’s understanding of ketogenesis, the process in which ketone bodies (KBs) are generated from fatty acid metabolism from acetyl-CoA in the liver. KBs include acetoacetate (ACA), D(-)3-hydroxybutyrate (D-βHB, β-HB), and acetone and the former two are shuttled into the citric acid cycle to generate ATP. Increased levels of ketones are able to “spare” glucose from being used in energetic tasks, and this action of KD has been implicated in underlying the therapeutic potential of KD on the brain (Zilberter and Zilberter, 2020).

#### Ketogenic diet relation to the brain

The brain is a highly metabolic instrument, and while glucose is its primary energy source, during times of starvation, ketone bodies fulfill up to 70% of the brain’s metabolic requirements (Owen et al., 1967). Plasma levels of KBs during prolonged fasting states range from 5.8 to 9.7 mM/l (Owen et al., 1967). The cerebral ability of ketone uptake is dependent on the plasma concentration of KBs and the level of monocarboxylic acid transporters (MCT) on the BBB (White and Venkatesh, 2011). It has been proposed that, while the adult brain is thought to have low levels of microvascular MCT expression on the BBB (Vannucci and Simpson, 2003), high levels of plasma KB correlate with high uptake of KBs in the brain. Therefore, MCT expression is suggested to be concentration-dependent on KBs (Hawkins et al., 1971; Leino et al., 2001; Vannucci and Simpson, 2003), evident in a study (Bentourkia et al., 2009) observing up to eight-fold increases in cerebral KB uptake during ketosis states. Ketones alone cannot adequately maintain the cerebral energetic needs so supplementation with proteins and minimal carbohydrates is often accompanied with a KD (White and Venkatesh, 2011).

#### Role of ketogenic diet in brain injury

The KD has been proposed to be beneficial and neuroprotective in chronic brain injury (Prins, 2008). The use of KD in providing alternative fuel to the brain during injury has been considered for decades; in a state of low cerebral blood flow, the energetic supply of glucose is compromised, and its metabolism is affected (O’Connell et al., 2005; Prins et al., 2013), which in turn compromises the metabolic needs of the brain. Exogenous supply of ketones can fulfill the energetic demands, especially considering the production and maintenance of ketones as well as its associated ATP production is suggested to be more efficient than glucose (Prins et al., 2004; Prins, 2008). Moreover, there is suggestive evidence in mouse models that the injured brain prefers ketone metabolism, as studies have observed increases in MCT2 (neuronal MCTs) transporters 6 and 24 h after TBI [178] and increases in MCT1 transporters within 24 h of ischemic injury (Tseng et al., 2003; Zhang et al., 2005). Recent studies have also highlighted that adequate and/or over-expression of MCTs likely has a role in alleviating poor stroke outcomes (Wang et al., 2011; Yu et al., 2021a). The temporal shift from glucose metabolism to ketone metabolism is unclear. Several studies (Zhang et al., 2013; Ma and Suzuki, 2019) on nutritional ketosis suggest that the shift from glucose metabolism to ketone metabolism takes time, but how long is unclear. Nonetheless, there is growing evidence to suggest that in an injured, energetically deprived state, cerebrum metabolism will switch to ketone metabolism to meet energetic demands.

**FIGURE 2:**
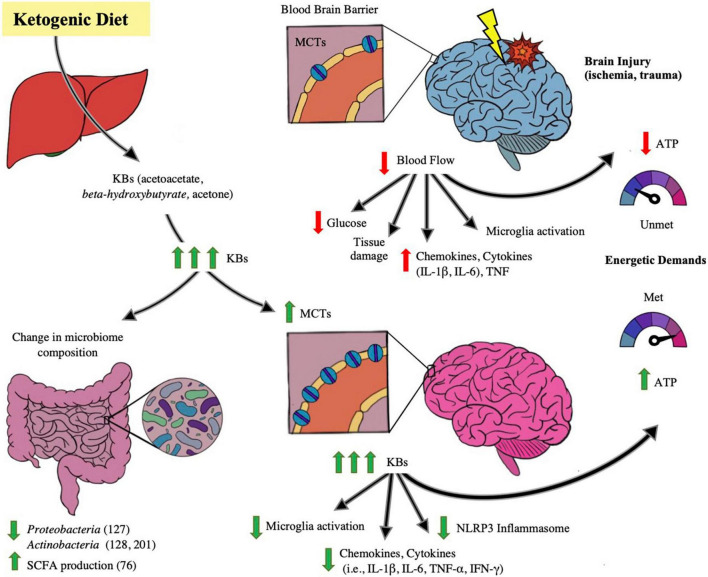
Impact of ketogenic diet ameliorating abnormalities in microbiome-gut-brain axis. The figure illustrates the effect of the ketogenic diet (KD) on alleviating brain injury and modulating the microbiome. With ischemic and traumatic acute brain injury, the energetic demands of the brain are compromised due to injury-provoked low cerebral blood flow and hence glucose supply to the brain- leading to lower production of ATP, exacerbation of brain parenchymal damage and propagating neuroinflammation, including microglial macrophage-like activity and cytokine (i.e., IL-1β, IL-6, TNF) release. Also depicted in the injured brain state is a baseline number of monocarboxylic acid transporters (MCTs) on the blood brain barrier (BBB), receptors that determine cerebral uptake of ketone bodies (KB). When KD is ingested and converted to its component ketone bodies (KB) through liver metabolism, the KBs lead to changes in the brain as well as the gut microbiome. In the microbiome, several studies note beneficial changes in taxa in both animal and human models, including a decrease in *Proteobacteria* (Medel-Matus et al., 2018), *Actinobacteria* (De Caro et al., 2019; Zilberter and Zilberter, 2020), and a rise in SCFA levels (Nagpal et al., 2019). In the brain, with increases of systemic KB supply, there is increase of synthesis of MCT receptors on the BBB, leading to enhanced cerebral uptake of KBs. KBs have been proposed to act as efficient alternative cerebral fuel, reinstating the energetic demands of the brain by increasing ATP production, as well as mitigating neuroinflammation by decreasing the pro-inflammatory activation of NLRP3 Inflammasome, and decreasing microglial activation and cytokine and chemokine release. There is growing evidence that KB has the potential of improving the injured and diseased brain states.

Inflammation is a hallmark in exacerbating neural disease and/or injury. Neuroinflammation is associated with the following changes in the brain: microglia activation, increased circulation of inflammatory markers such as chemokines, cytokines (such as IL-1β, IL-6) and necrosis factor (TNF), peripheral cell recruitment, and local tissue damage (O’Callaghan et al., 2008; Tremblay et al., 2011; Estes and McAllister, 2014; Woodburn et al., 2021). Means to mitigate the inflammation propagated by primary brain injury is vital in avoiding poor outcomes after brain injury, and KD’s role in doing so is depicted in Figure 2. Mechanisms of KD’s neuroprotective and anti-inflammatory affect has been proposed by Gough et al. (2021) to be of several means, including: KBs serving as alternative fuel for brain metabolism and thereby restoring energetic needs of the brain, induction of anti-inflammatory signaling pathways such as suppressing the NLRP3 inflammasome, reduction of oxidative stress by increasing the histone acetylation of FoxP3 leading to the reduced release of reactive oxygen species, and alterations to the gut microbiome. The KD and its production of ketones have been linked to mitigating acute and chronic neuroinflammation and injury.

#### Ketogenic diet interventions in chronic neurologic disorders

The use of KD and its induced state of ketosis to treat neurological disease dates back to over a century ago, where it was implemented as a treatment for pediatric epilepsy at the Mayo Clinic in 1921 (Wheless, 2008). Since then, research on the KD diets in relation to disease states have greatly increased in the last two decades. The efficacy and safety of achieving a state of ketosis in the study of neuronal injury in both animal and human trials is discussed in Table 2.

**TABLE 2:** Studies analyzing the safety and efficacy of KD with evidence of sustained ketosis.

Study	Study type	Disease	Intervention	Results	Measuring ketosis	Safety of KD
Animal models						
Streijger et al., 2013	C5 hemi-section rodent model	Spinal cord injury	KD 3:1 4 h following injury	The study associated KD to smaller spinal lesion sizes and improved ipsilateral forelimb movement, and, interestingly, when MCT was pharmacologically inhibited, the neuroprotective effect by KD was prevented	Blood Ketone concentrations (mmol/L), specially βHB, *via* Medisense Precision Xtra monitor. Rodents sustained ketosis for 12 weeks after injury.	Not addressed
Kong et al., 2017	C5-hemisection rodent model	Spinal cord injury	KD 7:1 2 weeks prior to injury	KD metabolite beta-Hydroxybutyrate administration can mitigate oxidative stress in spinal cord injury through KD’s suppression of class I histone deacetylases.	Measured blood ketone levels (βHB) – Two weeks prior to injury, ketone levels reached ∼2.8 mM, then decreased to ∼1.5 mM the day after SCI, and then slightly increased at 3 days after SCI. At 7 days post-injury, ketone level increased to pre-injury levels (∼2.8 mM).	Not addressed
Tai et al., 2008	Rodent model	Cardiac arrest induced cerebral ischemia	KD fat (78%), protein (10%), carbohydrate (2%), and inert components (10%).	KD prevented cardiac arrest-induced cerebral ischemic events, as well as neurodegeneration in the cerebellum and thalamic reticular nucleus.	Sustained ketosis for 25 days in KD fed rats was confirmed by colorimetric determination of the blood βHB level using a βHB LiquiColor kit	Not addressed
Shaafi et al., 2019	Stereotactic endothelin-1 (ET-1) MCA stroke model	Ischemic stroke	KD 4:1 + 3.5 cc/day MCT oil + 12 h fast before to induce ketosis quicker, given 3 days prior to stroke induction	Preconditioning KD significantly reduced motor dysfunction as measured by all motor-behavior function assessments.	KB levels measured prior to KD, and repeated on 2nd, 4th, 6th, 9th, 11th day of the study. KB levels were significantly higher than the other two groups from the second day and sustained consistently during the remainder of the study (3.39 ± 0.81 mmol/L, 0.31 ± 0.09 and 0.34 ± 0.07 mmol/L respectively, *P* < 0.001).	Not addressed
Ma et al., 2018	Young healthy mice	Neurovascular integrity	KD feed (not specified) over 16 weeks	At 16 weeks, KD fed mice had significant increases in cerebral blood flow, BBB *P*-glycoprotein channel transporter’s, reduced mTOR, and enhanced expression of endothelial nitric oxide syntheses. Changes in microbiota was noted – increases in *Akkermansia muciniphila* and *Lactobacillus* and decreases in *Desulfovibrio* and *Turicibacter.*	Measured blood ketone levels - KD fed mice had significantly higher (43%) ketone levels compared to control mice (*p* = 0.0004). Ketone and glucose levels were inversely related.	Not addressed
Ari et al., 2014	SOD1-G93A transgenic ALS mice	ALS	KD custom-diet with macronutrient profile similar to human prescribed KD. Started diet at 10 weeks old and continued until survival endpoint.	KD-fed mice had greater motor performance and extended survival time than controls fed a standard diet.	Blood collected from tail snip of glucose and βHB at survival endpoint. Blood βHB was over 50% higher in KD fed mice.	Not addressed
Zhao et al., 2006	SOD1-G93A transgenic ALS mice	ALS	KD feed based on known human formulation	KD has been associated with augmented motor unit survival and motor performance improvement.	KD fed mice showed > 3.5 elevation in the blood concentration of circulating ketone bodies (acetone, acetoacetate, and βHB) compared to standard feed animals (1056 ± 197 vs. 360 ± 43 μM, *p* = 0.012). The principle KB (78%) measured was is βHB.	Not addressed
Human studies						
Phillips et al., 2018, 2021; Taylor et al., 2018; White et al., 2020	RCT, crossover trial	Alzheimer’s Disease	Low fat diet versus ketogenic diet recipes which constituted 58% fat, 29% protein, 7% fiber, and 6% net carbohydrate by weight - 12 weeks intervention with 10 weeks washout period	Modified KD achieved sustained ketosis during KD period and cognitive performance improvement in AD Cooperative Study - Activities of Daily Living (ADCS-ADL) inventory, and Quality of Life in AD (QOL-AD) questionnaire in those with Alzheimer’s disease.	Patients who completed the ketogenic diet reached sustained physiological ketosis (12-week mean beta-hydroxybutyrate level: 0.95 ± 0.34 mmol/L).	Mild adverse effects – irritability, fatigue, sugar cravings. Irritability is a common adverse effect of AD [28].
Nagpal et al., 2019	Double-blind, cross-over, pilot RCT	Alzheimer’s disease	MMKD versus American Heart Association Diet (AHAD) - 6-week diet intervention with 6 weeks washout period.	MMKD increased abundance of *Enterobacteriaceae*, *Akkermansia*, *Slackia*, *Christensenellaceae* and *Erysipelotrichaceae* and decreased levels of *Bifidobacterium* and *Lachnobacterium.* MMKD had *minor* decreases in fecal lactate and acetate but increases SCFAs propionate and butyrate.	Blood ketone levels measured weekly to ensure compliance – 17 of 23 initiating the diets were included in the fecal analysis	No adverse effects or safety of KD was addressed
Nagpal et al., 2020	Randomized, double-blind, crossover, single-center pilot trial	Mild cognitive impairment (MCI)	MMKD versus American Heart Association Diet (AHAD) - 6-week diet intervention with 6 weeks washout period.	MMKD induced a broader effect on gut fungal (mycobiome) diversity in subjects with MCI compared to cognitively normal controls. MMKD on MCI also increased *Agaricus* and *Mrakia* while decreased *Saccharomyces* and *Claviceps* fungal taxons.	Not addressed	Not addressed
Taylor et al., 2018	Pilot RCT	Alzheimer’s disease	MCT supplemented KD aka “Very high-fat KD” (VHFKD) – 70% fat including MCT, 1:1 ketogenic ratio, 8 weeks	Study confirmed ketosis and cognitive improvement over the 3-month period, noted cognitive reversion to baseline during the washout phase, and justified safety of KD in AD.	Urine acetoacetate levels were measured daily by participants and βHB plasma levels were measured monthly. Study noted significant increases in βHB levels during the VHFKD period but urine results did not suggest consistent acetoacetate levels throughout the diet (60.6% of participants resulted some degree of acetoacetate in their urine)	MCT-associated diarrhea.
Phillips et al., 2018	Pilot RCT	Parkinson’s disease	Low-fat diet vs. KD. KD meal plan consisted of daily intake of 1,750 kcal per day composed of 42 g of fat, 75 g of protein, 246 g net carbohydrate, and 33 g of fiber.	PD patients adhering to KD diet sustained ketosis (measured *via* βHB) and had significantly decreased MDS-Unified Parkinson’s Disease Rating Scale scores and greater improvements in non-motor symptoms (cognitive impairment, fatigue, pain sensations) compared to patients fed a low-fat diet.	Measured βHB levels. Over 8 weeks, results saw significant differences between the diet groups in weekly mean bedtime blood glucose (low−fat group: 6.28 ± 0.73 mmol/L vs. ketogenic group: 5.70 ± 1.20 mmol/L; *P* = 0.001) and ketone (0.16 ± 0.05 mmol/L vs. 1.15 ± 0.59 mmol/L; *P* < 0.001) levels.	Minor exacerbation of tremor and/or rigidity - 50% of patients week 1–4 then reduced to 29% in weeks 5–8.
White et al., 2020	Phase II RCT intervention.	Acute brain injury – phase II intervention - enrolled within 48 h of admission	KD combo enteral feed *via* NG tube: KetoCall^®^ (4:1 Ratio, 88.7% fat, 8.3% protein, 3.1% CHO) + Protifar^®^ (protein feed 28 g/l to meet caloric goal)	The study noted that ketone levels increased with KD enteral feed and that KB levels correlated with KetoCal caloric intake. The study concluded the safety of KD tube feeds on adult brain injury (no acid/base changes, hypoglycemia, seizures) but did not note an impact of KD on cerebral hemodynamics.	Plasma KB levels of βHB and acetoacetate were measured. Study noted a significant increase of plasma KBs during the study period, with βHB levels rising from 0.24 ± 0.31 mmol/l to 0.61 ± 0.53 mmol/l (*p* = 0.0005) and acetoacetate levels increasing from 0.19 ± 0.16 mmol/l to 0.52 ± 0.40 mmol/l (*p* < 0.0001) over the study period. Sustained reduction of plasma glucose from day one until day six.	GI side effects, noted an increase in several hepatocellular and cholestatic enzymes

Recent research has shifted attention on the microbiome and how it can be connected to the neuroprotective effects of KD in brain injury and disease. An interesting study by Olson et al. (2018) observed KD protective effects against seizures; KD-fed mice subjected to microbiome depletion through antibiotic use or germ-free rearing saw increases in seizure frequency—no longer experiencing the protective effect of KD. When antibiotics were discontinued, the microbiome was restored and showed a reduced alpha diversity and enhanced KD associated *Akkermansia* and *Parabacteroides* that, combined, restored the KD defense against seizures. Furthermore, the study demonstrated the same KD-associated seizure effect in control mice that underwent a FMT from KD-fed mice (Olson et al., 2018). The study also noticed a correlation with KD-induced seizure protection on a systemic level such as increased GABA:glutamate hippocampal levels and decreased systemic gamma-glutamylated amino acids (Olson et al., 2018). This study was novel in its suggestion of microbes playing a role in influencing hippocampal excitability through regulation of peripheral metabolites with KD.

Several human studies have correlated microbial changes with KD and seizure alleviation. A study on children with drug-resistant epilepsy (DRE) saw, within 1 week of KD diet initiation, KD-associated changes to the microbiome included decreased alpha diversity, reduced composition of *Proteobacteria*, and enhanced composition of beneficial bacteria such as the phylum *Bacteroidetes* (Xie et al., 2017). Another study noted that 3-month KD use decreased levels of *Actinobacteria* and *Bifidobacterium* and increased levels of *Proteobacteria* (Lindefeldt et al., 2019) while another study observed that levels of *Actinobacteria* and *Firmicutes* were reduced relative to *Bacteroides* after 6 months of KD treatment in children with DRE (Zhang et al., 2018). A study by Zhang et al. (2018) saw variable responses to KD and correlated it to microbiome compositions; Interestingly, those that did not respond favorably to the KD had enhanced levels of *Alistipes*, *Clostridiales*, *Lachnospiraceae*, *Ruminococcaceae*, and *Rikenellaceae* compared to patients that responded well to KD (Zhang et al., 2018). A recent study observed a higher ketogenic ratio and diet compliance in DRE adult patients supplemented an Adkins diet with KetoCal^®^; however, the study did not notice augmented seizure reduction (McDonald et al., 2018). The effect of KD on adults with DRE is currently not clear (Martin-McGill et al., 2020) and warrants more study.

Beyond epilepsy, KD has been suggested to be therapeutic in mitigating neuroinflammatory processes. In a rodent model of Parkinson’s disease, KD was suggested to reduce microglia activation and suppress inflammatory factors such as IL-1β, IL-6, and TNF-α (Yang and Cheng, 2010). KD therapeutic use in human PD has suggested safety and efficacy, with a recent pilot RCT observing greater improvements in non-motor symptoms such as fatigue and pain sensation in those fed the KD diet for 8 weeks compared to a low-fat diet (Phillips et al., 2018). The therapeutic tie between the microbiome and KD in PD has not be described. In ALS, KD has been associated with augmented motor unit survival, motor performance improvement, and extended survival time in rodent models (Zhao et al., 2006; Ari et al., 2014).

The role of KD in Multiple Sclerosis has been described in rodent and human modals. In a rodent model of MS, KD was associated with the downregulation of CD4 + cells, microglia, and expression of cytokines (IL-1β, IL-6, TNF-α, IL-12, IL-17) and chemokines (IFN-γ, MCP-1, MIP-1α, MIP-1β) (Kim et al., 2012). KD has been implicated in providing therapeutic potential in MS rodent models, such as the potential of axonal regeneration (Storoni and Plant, 2015), however clinical evidence in human trials is lacking (Bahr et al., 2020). In a human study of 25 MS patients, quantification of microbiome signatures defined significant reductions in colonic bacteria, namely *Roseburia*, *Bacteroides*, and *Faecalibacterium prausnitzii* (Swidsinski et al., 2017); however, with KD, microbiome abundance and diversity improved. In KD-fed MS patients the study observed increases in colonic composition at week 12, and improved diversity and abundance values (except for *Akkermansia*) comparable to healthy controls by week 24 (Swidsinski et al., 2017). In a more recent study on the use of long-term (18 months) KD therapy, the authors cited limited clinical evidence of KD improving MS disease severity (Bahr et al., 2020). Further study of KD therapy for MS disease severity as well as microbiome improvement is warranted.

A recent study on mild cognitively impaired (MCI) patients attributed the modified-KD to changes in the microbiome signatures and increases in SCFA production (Nagpal et al., 2019). Bacterial composition in KD-fed MCI patients was noteworthy for increases in *Enterobacteriaceae, Akkermansia, Slackia, Christensenellaceae*, and *Erysipelotrichaceae* while reductions in abundance of *Bifidobacterium* and *Lachnobacterium* were noted. Researchers linked particular microbiome changes to improved AD biomarkers, such as amyloid beta and tau proteins, in the CSF (Nagpal et al., 2019). A recent crossover study with AD patients noted the safety and efficacy of KD in inducing sustained ketosis and leading to cognitive performance improvement (Phillips et al., 2021). The safety of using KD in AD patients was further described, with another study utilizing a KD supplemented with medium-chain triglycerides (MCTs) noting MCT-associated diarrhea in 50% of patients, but reporting no changes in blood glucose levels, insulin, liver function tests, renal functioning, electrolyte balances, body mass index, bone mass or EKG, concluding the safety of this high-fat diet in AD for at least 3 months (Taylor et al., 2018). Another recent study observed the effect of KD on neurovascular integrity and its relationship with microbiome changes in mice (Ma et al., 2018). The study noted changes in microbiota composition which included increased abundance of beneficial *Akkermansia muciniphila* and *Lactobacillus* and decreased abundance of inflammatory *Desulfovibrio* and *Turicibacter*. In addition, after 16 weeks of KD feed, mice had significant increases in cerebral blood flow and *P*-glycoprotein channel transporters on the BBB to encourage the clearance of Amyloid beta proteins (Ma et al., 2018). The study of KD in healing chronic brain injury states is promising in animal models and has been extended for therapeutic use in humans. Studies in chronic injury states have provided a basis of efficacy and safety of KD and have encouraged study into the interplay of acute brain injury and the microbiome.

#### Ketogenic diet interventions in acute brain injuries

While limited, the study of KD and its component KBs in neuroinflammation is growing. Several studies have suggested a significant role by the ketone body β-HB in mitigating neuroinflammation. In an animal model looking at the acute response of KD on stress, it was noted β-HB caused the acute production of mitochondrial H_2_O_2_ and 4-hydroxy-2-nonenal (4-HNE), suggestive of activating the Nrf2 pathway, the primary cellular response pathway (Milder et al., 2010). Similarly, a study isolated β-HB in its direct role in preventing the NLRP3 inflammasome, which in turn suppressed the release of inflammatory cytokines IL-1β and IL-18 by caspase-1 in human monocytes (Youm et al., 2015). Suppression of NLRP3 inflammasome is considered significant and, in recent years, has gained attention in being utilized as a target in mitigating neuroinflammatory disease (Shao et al., 2018). More recently, in a fish model, β-HB was observed to inhibit LPS-induced inflammation seemingly by up-regulating anti-inflammatory genes such as NF-κBIA (Qiao et al., 2020).

A few animal models investigate the KD effect on acute CNS injury. While traumatic spinal cord injury (SCI) is not addressed in this review due to the scope of brain injury, there are several parallels to the pathophysiology of TBI with SCI (Jogia and Ruitenberg, 2020). In rodent SCI models, the KD intervention was associated with smaller spinal lesion sizes and improved ipsilateral forelimb movement (Streijger et al., 2013) and reduction of oxidative stress (Kong et al., 2017). In stroke models, KD-preconditioning significantly reduced motor dysfunction after stroke (Shaafi et al., 2019). Such promise in improved stroke outcomes has prompted study in human studies.

Human studies of KD and acute brain injury are very limited. Recently, a Phase II randomized control trial was done in the setting of acute brain injury with KD enteral administration (White et al., 2020). The study included patients with ischemic stroke, subarachnoid hemorrhage, and TBI and noted that ketone levels increased with KD enteral feed and that KB levels correlated with KetoCal^®^ caloric intake, but no impact of KD on cerebral hemodynamics was observed (White et al., 2020). The study concluded the safety of KD tube feeds on adult brain injury, with no acid/base changes, hypoglycemic emergencies, or seizure development (White et al., 2020). KetoCal^®^ and KetoVie^®^ are two commercially available formulas for dietary medical management of DRE epilepsy treatment and other KD-benefiting conditions, with both products consisting of a liquid diet with a 4:1 ketogenic ratio formula. Long-term compliance with KD can be challenging considering patients may opt to deter from the regimented diet for more appetizing foods. In the realm of acute brain injury, the use of Ketogenic feeds can be very promising, considering issues with compliance can be regulated in a hospital setting. While the studies mentioned prior noted effects of KD on chronic brain injury and correlated them with microbiome changes, studies in the context of KD therapy for acute brain injury do not cite any microbiome observations. Given the infancy of the study of the KD-microbiome connection, especially in the context of acute brain injury, such a gap is expected but warrants further trial.

## Conclusion/future directions

Taken together, the microbiome is affected by neurodegenerative conditions as well as brain injury states. While the specific changes to the microbiomes of patients suffering different neurological disorders are varied, a common theme is presented, in both acute and chronic brain injury: pathogenic and opportunistic bacteria like *Enterobacteriaceae and* Proteobacteria are enhanced why mutualistic and beneficial taxa such as *Faecalibacterium* and SCFA-producing bacteria are relatively depleted. It can be argued that the presence of gut dysbiosis and abundance of opportunistic bacteria is linked to the exaggeration of neuroinflammation, delay in functional recovery, and aggravation of tissue damage. With our growing understanding of the link between brain injury and dysbiosis, the KD provides a promising opportunity in meeting the unmet metabolic, energetic, and immunologic demands of the brain in an acute and chronic state of injury and inflammation.

The safety and efficacy of KD in human subjects has been extensively studied and confirmed in chronic neurological disorders (Walczyk and Wick, 2017; Gough et al., 2021; Yurista et al., 2021; Pietrzak et al., 2022; Zhu et al., 2022). While safety in chronic use has been established and confirmed with some acute injury studies, there is a relative lack in understanding the therapeutic potential of KD in acute injury states such as ischemic stroke, cerebral hemorrhages, and TBI. Moreover, the impact of KD on the microbiome and how it interacts with the pathogenesis and/or outcomes of various neurological disorders warrants better understanding. Substantial efforts should be made in order to elucidate the potential of high fat diets like KD in modulating the gut microbiome and lending itself to the establishment of new therapeutic targets.

## Author contributions

MK was involved in the literature search of the current state of science to be discussed in the review as well as consolidating ideas and writing up the greater majority of the review, created the tables and as well as the original artwork of the figures. AKS was MK’s primary mentor along the review writing process, guided the direction of MK’s research, provided frequent feedback, created the format and flow of the article, and edited multiple draft phases. NA provided meaningful edits in the draft phases of the review and contributed some information into the review. HY directed the formatting and scope of the review and provided meaningful feedback in the writing process. SJ, JG, KD, and BH provided meaningful feedback during the editing phase. All authors contributed to the article and approved the submitted version.
